# Supplementary material for: Burnout syndrome among frontline doctors of secondary and tertiary care hospitals of Bangladesh during COVID-19 pandemic
Source: PLoS One. 2022 Nov 22;17(11):e0277875. doi: 10.1371/journal.pone.0277875 (PMC9681110; doi:10.1371/journal.pone.0277875)
Supplement: S1 Table — (DOCX) [file pone.0277875.s005.docx]

**S1 Table. Supplementary Tables: Post-hoc analysis**

Post hoc analysis revealed that the mean EE score was significantly higher in doctors of the Obs. & Gynae department than the doctors in the Medicine department (p=0.004).

| Dependent Variable: EE | | | | | | | |
| --- | --- | --- | --- | --- | --- | --- | --- |
|  | (I) Duty_place | (J) Duty_place | Mean Difference (I-J) | Std. Error | Sig. | 95% Confidence Interval | |
|  |  |  |  |  |  | Lower Bound | Upper Bound |
| Medicine | Obs&Gynae | Medicine | 3.976^*^ | 1.146 | **.004** | 1.00 | 6.95 |
|  |  | ICU | 2.229 | 1.291 | .314 | -1.12 | 5.58 |
|  |  | Surgery& emergency | 1.132 | 1.274 | .811 | -2.17 | 4.44 |
|  | Medicine | Obs&Gynae | -3.976^*^ | 1.146 | .004 | -6.95 | -1.00 |
|  |  | ICU | -1.747 | 1.146 | .425 | -4.72 | 1.23 |
|  |  | Surgery& emergency | -2.844 | 1.126 | .060 | -5.77 | .08 |
|  | ICU | Obs&Gynae | -2.229 | 1.291 | .314 | -5.58 | 1.12 |
|  |  | Medicine | 1.747 | 1.146 | .425 | -1.23 | 4.72 |
|  |  | Surgery& | -1.097 | 1.274 | .825 | -4.40 | 2.21 |
|  | Surgery&  emergency | Obs&Gynae | -1.132 | 1.274 | .811 | -4.44 | 2.17 |
|  |  | Medicine | 2.844 | 1.126 | .060 | -.08 | 5.77 |
|  |  | ICU | 1.097 | 1.274 | .825 | -2.21 | 4.40 |
| *. The mean difference is significant at the 0.05 level. | | | | | | | |

Post hoc analysis revealed that the mean DP score was significantly higher in doctors in the ICU department than the doctors in the Medicine department (p=0.046)

| **Multiple Comparisons** | | | | | | | |
| --- | --- | --- | --- | --- | --- | --- | --- |
| Dependent Variable: DP | | | | | | | |
|  | (I) Duty_place | (J) Duty_place | Mean Difference (I-J) | Std. Error | Sig. | 95% Confidence Interval | |
|  |  |  |  |  |  | Lower Bound | Upper Bound |
| Tukey HSD | Obs&Gynae | Medicine | 1.388 | .615 | .112 | -.21 | 2.98 |
|  |  | ICU | -.229 | .693 | .988 | -2.03 | 1.57 |
|  |  | Surgery& emergency | .419 | .683 | .928 | -1.35 | 2.19 |
|  | Medicine | Obs&Gynae | -1.388 | .615 | .112 | -2.98 | .21 |
|  |  | ICU | -1.617^*^ | .615 | **.046** | -3.21 | -.02 |
|  |  | Surgery& emergency | -.969 | .604 | .379 | -2.54 | .60 |
|  | ICU | Obs&Gynae | .229 | .693 | .988 | -1.57 | 2.03 |
|  |  | Medicine | 1.617^*^ | .615 | .046 | .02 | 3.21 |
|  |  | Surgery& | .648 | .683 | .779 | -1.13 | 2.42 |
|  | Surgery&  emergency | Obs&Gynae | -.419 | .683 | .928 | -2.19 | 1.35 |
|  |  | Medicine | .969 | .604 | .379 | -.60 | 2.54 |
|  |  | ICU | -.648 | .683 | .779 | -2.42 | 1.13 |
| *. The mean difference is significant at the 0.05 level. | | | | | | | |

Post hoc analysis revealed that the mean DP score was significantly higher in doctors engaged in COVID duty than the doctors with mixed ward duty (p=0.008)

| **Multiple Comparisons** | | | | | | | |
| --- | --- | --- | --- | --- | --- | --- | --- |
| Dependent Variable: deperso_T | | | | | | | |
|  | (I) duty_type | (J) duty_type | Mean Difference (I-J) | Std. Error | Sig. | 95% Confidence Interval | |
|  |  |  |  |  |  | Lower Bound | Upper Bound |
| Tukey HSD | COVID | Non-COVID | 1.229 | .845 | .316 | -.77 | 3.23 |
|  |  | Mixed | 1.407^*^ | .467 | **.008** | .30 | 2.51 |
|  | Non-COVID | COVID | -1.229 | .845 | .316 | -3.23 | .77 |
|  |  | Mixed | .179 | .833 | .975 | -1.79 | 2.15 |
|  | Mixed | COVID | -1.407^*^ | .467 | .008 | -2.51 | -.30 |
|  |  | Non-COVID | -.179 | .833 | .975 | -2.15 | 1.79 |
| *. The mean difference is significant at the 0.05 level. | | | | | | | |
