# Supplementary material for: Burnout syndrome among frontline doctors of secondary and tertiary care hospitals of Bangladesh during COVID-19 pandemic
Source: PLoS One. 2022 Nov 22;17(11):e0277875. doi: 10.1371/journal.pone.0277875 (PMC9681110; doi:10.1371/journal.pone.0277875)
Supplement: Attachment — Submitted filename: Response to Reviewer.doc [file pone.0277875.s006.doc]

Dear Editor

PLOS ONE,

We appreciate the feedback from the Editor and reviewers and their helpful suggestions for further improving our manuscript. We have carefully considered all the valuable comments and made revisions accordingly. We have revised the entire manuscript text to enrich and avoid grammatical errors and rewrite it in sound English. Text changes are marked in color in the attached revised manuscript. We also formatted the revised manuscript according to the journal style. We satisfactorily responded to all the issues raised and are looking forward to your reconsideration of our manuscript.

We thank the referees for evaluating the manuscript and suggestions that substantially improved the manuscript's quality. In the following, we briefly cite the comments made by the referees and state how we responded to them.

We would be happy to include the manuscript to of unique collection "Burnout Syndrome Among Frontline Doctors of COVID Hospitals During COVID-19 Pandemic, Bangladesh."

With best regards,

Dr. Fahmida Rashid

Department of Obstetrics & Gynecology

Chittagong Medical College, Chattogram, Bangladesh

E-mail: dr.fahmidaswati@gmail.com

**Response to Staff Editor Comments:**

1. Please ensure that your manuscript meets PLOS ONE's style requirements, including those for file naming. The PLOS ONE style templates can be found at

<https://journals.plos.org/plosone/s/file?id=wjVg/PLOSOne_formatting_sample_main_body.pdf>

<https://journals.plos.org/plosone/s/file?id=ba62/PLOSOne_formatting_sample_title_authors_affiliations.pdf>

**Response:** We have formatted the revised manuscript and named the files according to the journal style according to the suggestion.

2. In your Data Availability statement, you have not specified where the minimal data set underlying the results described in your manuscript can be found. PLOS defines a study's minimal data set as the underlying data used to reach the conclusions drawn in the manuscript and any additional data required to replicate the reported study findings in their entirety. All PLOS journals require that the minimal data set be made fully available. For more information about our data policy, please see <http://journals.plos.org/plosone/s/data-availability>.

"Upon re-submitting your revised manuscript, please upload your study's minimal underlying data set as either Supporting Information files or to a stable, public repository and include the relevant URLs, DOIs, or accession numbers within your revised cover letter. For a list of acceptable repositories, please see [http://journals.plos.org/plosone/s/data-availability#loc-recommended-repositories](http://journals.plos.org/plosone/s/data-availability#loc-recommended-repositories). Any potentially identifying patient information must be fully anonymized.

Important: If there are ethical or legal restrictions to sharing your data publicly, please explain these restrictions in detail. Please see our guidelines for more information on what we consider unacceptable restrictions to publicly sharing data: [http://journals.plos.org/plosone/s/data-availability#loc-unacceptable-data-access-restrictions](http://journals.plos.org/plosone/s/data-availability#loc-unacceptable-data-access-restrictions). Note that it is not acceptable for the authors to be the sole named individuals responsible for ensuring data access.

We will update your Data Availability statement to reflect the information you provide in your cover letter

**Response:** Data Availability Statement: The analyzed data is all in the manuscript. However, the dataset of this study is available upon request from the corresponding author.

**3.**  We note you have included a table to which you do not refer in the text of your manuscript. Please ensure that you refer to Table 5 in your text; if accepted, production will need this reference to link the reader to the Table.

**Response:** According to the suggestion, we have referred the Table 5 in the text of the results section (Page 12, line 168).

**Responses to Reviewer #1**

1. The paper requires some editing, as it is not written in sound English and should not be accepted in its current form. The author(s) may get the manuscript revised by either a native English speaker or someone who excels in the English language for this purpose.

**Response:** Thanks for the comments. According to the suggestion, we have taken help from a colleague of the English Department of a Local University and a senior colleague experienced in scientific writing from our institute. With their use, we have revised the entire manuscript to avoid language and grammatical errors.

2. The introduction section is well-defined with adequate references.

**Response:** Thank you, we appreciate the comments.

3. The materials and methods section is described in detail. However, the results section has not been interpreted properly.

**Response:** Thank you for your valuable comments. According to your suggestion, we revised the results section and interpreted the results elaborately.

4. There are places, although not many, in the discussion section where relevant references should have been provided to strengthen the findings. Besides, a more comprehensive discussion on what needs to be done for an improved outcome could enhance the value of the work.

**Response:** Thanks for the remark, and we agree with that. We included few additional references to strengthen the findings.

**Responses to Reviewer #2**

**Reviewer #2**: Thank you for allowing me to review this manuscript.
The authors address a relevant and topical issue, such as the impact working on the front line against covid has on health professionals.
However, the manuscript can be improved. The international literature on burnout in health professionals is extensive, as is the literature referring specifically to burnout during the COVID pandemic. For this article to be publishable, the authors must still make an important effort to go deeper both in the background and in the discussion. To do this, they must substantially expand the international literature they handle and reference (in the current version, only 14 references, of which six are more than ten years old).
The methodology and presentation of results are a longitudinal descriptive study, and the results are presented adequately. However, they could have been enriched by adding additional variables (perceived stress, workload, quality of life ... .), which would allow a greater depth in the discussion and conclusions.
There are small errors about the sample size (in the method, there are 165 professionals, and in results, it is said that 168).
For all of them, I believe that this manuscript does not meet the minimum requirements for publication in its current version. However, I think that the authors should continue to delve into this topic of such interest.

**Response:**

According to your suggestion, we made an effort to review related recent international literature on burnout in health professionals. After reviewing those articles, the background and discussion sections were revised and edited accordingly.

Regarding your suggestion of adding some additional variables, we will keep it in our mind for our future work.

The sample size was 168, corrected in the methods section (Page 5, line 94). There are minor errors about the sample size (in the method, it is said that there are 165 professionals, and in results, it is said that 168).Thanks for marking it.
