# Supplementary material for: Burnout syndrome among frontline doctors of secondary and tertiary care hospitals of Bangladesh during COVID-19 pandemic
Source: PLoS One. 2022 Nov 22;17(11):e0277875. doi: 10.1371/journal.pone.0277875 (PMC9681110; doi:10.1371/journal.pone.0277875)
Supplement: Attachment — Submitted filename: Rebuttal letter t (1).doc [file pone.0277875.s007.doc]

**Responses to Reviewer #2:**

Reviewer #2: All comments have been addressed

2. Is the manuscript technically sound, and do the data support the conclusions?

The manuscript must describe a technically sound piece of scientific research with data that supports the conclusions. Experiments must have been conducted rigorously, with appropriate controls, replication, and sample sizes. The conclusions must be drawn appropriately based on the data presented.

Reviewer #2: Yes

3. Has the statistical analysis been performed appropriately and rigorously?

Reviewer #2: Yes

4. Have the authors made all data underlying the findings in their manuscript fully available?

Reviewer #2: Yes

5. Is the manuscript presented in an intelligible fashion and written in standard English?

PLOS ONE does not copyedit accepted manuscripts, so the language in submitted articles must be clear, correct, and unambiguous. Any typographical or grammatical errors should be corrected at revision, so please note any specific errors here.

Reviewer #2: Yes

6. Review Comments to the Author

Reviewer #2: The authors have made a great effort to answer the questions raised in the previous review, and the manuscript has improved substantially.
I recommend that authors cite or reference the article on the Bangladeshi version of the Maslach questionnaire (in addition to the original citation already included).
I also recommend reviewing the English, especially in the introduction

**Answer:** Thank you, we appreciate the comments. About your suggestion regarding TheMaslach Burnout Inventory, In our study, we have used Maslach Burnout Inventory for Human Services survey (MBI- HSS) –English version as all the respondents were physicians whose medium of instruction was English. It was easy for them to respond to the inventory appropriately.

The English of the article was addressed thoroughly by a native English-speaking person as per your suggestion.

7. PLOS authors have the option to publish the peer review history of their article ([what does this mean?](https://journals.plos.org/plosone/s/editorial-and-peer-review-process#loc-peer-review-history)). If published, this will include your full peer review and any attached files.


If you choose "no," your identity will remain anonymous but your review may still be made public.


Do you want your identity to be public for this peer review? For information about this choice, including consent withdrawal, please see our [Privacy Policy](https://www.plos.org/privacy-policy).

Reviewer #2: No
