# Supplementary material for: Burnout syndrome among frontline doctors of secondary and tertiary care hospitals of Bangladesh during COVID-19 pandemic
Source: PLoS One. 2022 Nov 22;17(11):e0277875. doi: 10.1371/journal.pone.0277875 (PMC9681110; doi:10.1371/journal.pone.0277875)
Supplement: Attachment — Submitted filename: Reveiwer comment on BOS.docx [file pone.0277875.s008.docx]

**Comments for authors**

Reviewer Comments for a research entitled: Burnout Syndrome among Frontline Doctors of COVID Hospitals during COVID-19 Pandemic, Bangladesh

1. Line number 1; the title of the research need to be editing that <<doctors of COVID Hospitals>> is not make sound so it is better to rewrite as: Burnout Syndrome among Frontline Doctors Working in COVID Hospitals during COVID-19 Pandemic, Bangladesh. But as you write in the abstracts section of the manuscript, the study was conducted in secondary and tertiary levels of hospital. Therefore, the title should be modified regarding this types of hospital rather than saying COVID hospital.
2. In the abstract , Method section as the title indicated that doctors working in COVID hospital or treated COVID patients why you include doctors working in <<non- COVID patients>> this may make inconclusive result regarding to COVID related BOS. The other concern in this section is you should add methods of data analysis and strength and significance of predictor variable expression.
3. In abstract, result section line number 19; you should modified or rewrites << rather saying entire sample>> you should use appropriate words like you can say << was found in all study participants>>. In line number 20-21 needs rewrite example; Prevalence of high EE, high DP, and reduced PA were observed in 58.3%, 98.2%, and 97% of study participants, respectively.

Line 22 ,<< female sex>> in this regard saying<< female>> is enough as it is self explanatory but you can write as <<Being female rather than saying female sex>>

In conclusion section, line number 26-28 it is better to add conclusion about which risk factors associated with the three domains of BOS by narrating.

4. From line number 32-36 needs rewrite and synthesis example << exceed this far>> for example to exceed far this.

This literature review section in general needs rewriting and grammatical editing

5. The Aim of the study was to assess BOS for doctors working in COVID 19, but in method section you take study participants from doctors’ work in non-COVID19 hospitals (line number 88-91). The 89 doctors working in these hospitals either provide services exclusively to COVID-19 patients, to 90 non-COVID-19 patients, or a mixture of COVID-19 and non-COVID-19 patients (termed 91 ‘mixed’ in this study) following the principles of isolation and quarantine. Therefore, how do you conclude the level of BOS for doctors working in COVID -19 hospitals for the future out break intervention for prevention of BOS?
6. For line number 93, you wrote about consent, which types of consent you obtained from the study participants.
7. Line number 97, in your data collection tool the first component is about socio –demographic and workplace details. Do you incorporate workplace factors for burn out syndrome like demand –control model (job control and job demand) model to see the effect of workload and workers capability to accomplished it.
8. Is the tool is validated in your research context
9. Line number 113-118, methods of data analysis, why do you use two methods of data analysis (ANOVA and binary logistic regression). In the use of ANOVA, do you see where is the mean difference of Burn out syndrome in the three domains of Dp, EE and PA
10. Line number 170 - 171 chi square test , High Burnout in all three Domains and demographic and Job related Characteristics you try to show the significant variable with that of in all domains of burn out in the table provided by itself the table should be mixed or prepared in one table with the regression analysis you used to determine independent factors with burn out syndrome meaning that :
1. In Chi square test you can get	cells for	burn out and for non- burn out for each categories interested variable and used to create two by two tables. From this to calculate crud odds ratio manually to check correctness of	odds ratio obtained by	statically analysis.
2. To determine independent factors with burn out syndrome in all the three models first you should determine each variable in binary logistic regression model ( one independent variable with outcome variable) and if you get p- value <0.2 or 0.25) and you can consider these variable is candidate for multivariable logistic regression analysis.
3. Report adjusted odds ratio rather than crude odds ratio, in this way create one table that consists all information as follows

| Variable | Burn out | | 95%CI Crude odds ration | 95%CI Adjusted odds ratio | p- value |
| --- | --- | --- | --- | --- | --- |
|  | Yes | No |  |  |  |
|  |  |  |  |  |  |

11. In table six the reference category should be written in number rather than words

and indicate it in footnotes

12. Line number 202-207, you only discuss your research prevalence is higher than other studies. Therefore, the prevalence of burn out	in your study should be discussed with other studies in three ways	1. Similarity with other studies 2. Higher than other studies	3. Lower than other studies with relevance reference citation.
13. What is over all prevalence of burn out syndrome after sum up of the three domains(with confidence intervals)
1. In general, the research article is very important to provide essential information regarding health care workers level of burn out syndrome during outbreak management to develop an intervention.
2. Grammer editing is needed
3. Rewrite or resynthesis of paragraphs is more needed
4. Even if including non- COVID19 ward study participants raised as limitation of the research and it is better rewrite by showing the difference of prevalence of burn out syndrome between COVID and non-COVID 19 ward study participants to appreciate the level of burn out syndrome between them.
