# Supplementary material for: Burnout syndrome among frontline doctors of secondary and tertiary care hospitals of Bangladesh during COVID-19 pandemic
Source: PLoS One. 2022 Nov 22;17(11):e0277875. doi: 10.1371/journal.pone.0277875 (PMC9681110; doi:10.1371/journal.pone.0277875)
Supplement: Attachment — Submitted filename: Response to Reviewers.docx [file pone.0277875.s010.docx]

**Responses to Reviewer's Questions**

**Reviewer #2: (**No Response)

**Reviewer #3:** Dear authors, your paper is enhanced significantly. However, minor issues still need more clarification before accepting this paper. English language editing is recommended.

**Abstract**

- conclusion part has to be written again. It's not a repeat of what in your results. Give a broad image of burnout during such crises among doctors. The implications for medicine must also be addressed.

**Introduction**

- Again, please set all references before the full stop dot.

- Add one paragraph related to your demographic factors.

**Methods**

- How did you collect your data by whom, and at what time, did they sign a consent form.... These issues need to be clarified.

Post hoc test: Which one??? Please add. Tukey, Sheffie....

**Results**

- In Table 5, why do you mention 2p-values.. Some of the p-values are significant without marks.

- Again, please change the word (sex) to gender in the whole of your manuscript.

The discussion part is improved significantly

Please return to the style of PLOS ONE reference guidelines, Some papers you added DOI, some references you add a month of publication.

Please double-check all references.

Good luck.

**Response to Reviewer #3 Comment:**

-Tried my best to improve the editing of the English language.

**In abstract:**

-Conclusion part of abstract rewritten according to your suggestion. Implication for medicine addressed.

Introduction:

- All references are set before the full stop dot.

- One paragraph is added related to my demographic factors.

**Methods:**

- How data was collected, by whom, at what time, and whether they signed a consent form. - These issues be clarified in the methods and material section under the heading-‘settings and participants'.

-Type of Post hoc analysis is added to results and discussion(Tukey HSD-Mentioned in Line 185 & 188).

**Results:**

-In table 5, 2 p-values were mentioned---One is for univariate analysis and an unadjusted odds ratio, and the other for the multivariate analysis-adjusted odds ratio. The significant p-values are marked by the star a (*) mark.

- The word 'sex' changed to gender in the whole manuscript.

-Double-checked references according to PloS one guideline and did necessary modifications.

**Reviewer #4: Comments for authors**

**1.** In the method section from line number 85 says the study was conducted from June to August 2020? Is it data collection period or the research is done with this time bound, if it is for data collection period I think it is so long 3 months for cross-sectional study data collection.

**Answer:** Thank you very much. Because of frontline doctors' lockdown and quarantine policy, it wasn't easy to research the doctors for data collection. It took three months to collect and compile data from two institutes.

**2.** In the result section what was your criteria to categorize the age of your study participants line number 144(socio-demographic characteristics)?

**Answer:** Categorization of the age of respondents into different groups based on their academic year(1st-3rd and 4th-5th year) is described in the result section.

**3.** In your data collection tool, the first component is about socio –demographic and workplace details. Do you incorporate workplace factors for burn out syndrome like demand –control model (job control and job demand) model to see the effect of workload and workers capability to accomplished it.

**Answer**: In our data collection tool, in the first component, only socio-demographic profiles were included, and workplace details were not incorporated. So, in the current manuscript, erroneously mentioned words related to workplace details are discarded.

**4.** Line number 175, High Burnout in All Three Domains and Demographic and Job-related and in it is regression table the category of <> were not have AOR why it cannot candidate for multivariable regression model and it is unusual even the change of odds ratio should be seen from bi variable to multivariable model, so it is better to see again it.

**Answer: In table -5(**High burnout in all three domains in demographic and demographic and Job-related characteristics) - only the variables with p<0.2 in bivariate analysis were included in the multivariable analysis.

The change of OR is rechecked according to your suggestion, and the results are the same.

5. In the discussion section after starting the discussion section with some statements that indicate the problem and next discussed the main finding of the your study therefore line number 198 -205 is part of you background or statement of the problem and it is better avoid it .

**Answer:** Correction done as per your suggestion.

The paper is very important to provide essential information regarding health care workers level of burn out syndrome during outbreak management to develop an intervention.

**Answer:**

Thank you.

1. **Please ensure that you refer to Table 6 in your text as, if accepted, production will need this reference to link the reader to the Table.**

**Answer:** Table 6 referred to text according to your suggestion.
