# Supplementary material for: Burnout syndrome among frontline doctors of secondary and tertiary care hospitals of Bangladesh during COVID-19 pandemic
Source: PLoS One. 2022 Nov 22;17(11):e0277875. doi: 10.1371/journal.pone.0277875 (PMC9681110; doi:10.1371/journal.pone.0277875)
Supplement: Attachment — Submitted filename: Rebuttal Letter.DOCX [file pone.0277875.s011.DOCX]

Journal Requirements:

Please review your reference list to ensure that it is complete and correct. If you have cited papers that have been retracted, please include the rationale for doing so in the manuscript text, or remove these references and replace them with relevant current references. Any changes to the reference list should be mentioned in the rebuttal letter that accompanies your revised manuscript. If you need to cite a retracted article, indicate the article's retracted status in the References list and also include a citation and full reference for the retraction notice.

**Answer:** Reviewed reference list. There are no retracted references at present.No change has been made in the reference list apart from the style of reference writing.

**Reviewer's Responses to Questions**

**Comments to the Author**

1. If the authors have adequately addressed your comments raised in a previous round of review and you feel that this manuscript is now acceptable for publication, you may indicate that here to bypass the "Comments to the Author" section, enter your conflict of interest statement in the "Confidential to Editor" section, and submit your "Accept" recommendation.

Reviewer #3: All comments have been addressed

Reviewer #4: (No Response)

2. Is the manuscript technically sound, and do the data support the conclusions?

The manuscript must describe a technically sound piece of scientific research with data that supports the conclusions. Experiments must have been conducted rigorously, with appropriate controls, replication, and sample sizes. The conclusions must be drawn appropriately based on the data presented.

Reviewer #3: Yes

Reviewer #4: Yes

3. Has the statistical analysis been performed appropriately and rigorously?

Reviewer #3: Yes

Reviewer #4: Yes

4. Have the authors made all data underlying the findings in their manuscript fully available?

The [PLOS Data policy](http://www.plosone.org/static/policies.action#sharing) requires authors to make all data underlying the findings described in their manuscript fully available without restriction, with rare exception (please refer to the Data Availability Statement in the manuscript PDF file). The data should be provided as part of the manuscript or its supporting information, or deposited to a public repository. For example, in addition to summary statistics, the data points behind means, medians and variance measures should be available. If there are restrictions on publicly sharing data—e.g. participant privacy or use of data from a third party—those must be specified.

Reviewer #3: Yes

Reviewer #4: Yes

5. Is the manuscript presented in an intelligible fashion and written in standard English?

PLOS ONE does not copyedit accepted manuscripts, so the language in submitted articles must be clear, correct, and unambiguous. Any typographical or grammatical errors should be corrected at revision, so please note any specific errors here.

Reviewer #3: Yes

Reviewer #4: No

**Answer:** According to your prior suggestion, the manuscript was checked and improvised by a native English-speaking person(as I mentioned earlier). And any typographical or grammatical errors corrected, reformed as much as possible.

6. Review Comments to the Author

Please use the space provided to explain your answers to the questions above. You may also include additional comments for the author, including concerns about dual publication, research ethics, or publication ethics. (Please upload your review as an attachment if it exceeds 20,000 characters)

Reviewer #3: Congratulations. All of my comments have been added. My best wishes to authors for their effort. Great work

Reviewer #4: Dear editors , Thank you giving this opportunity to review this research paper the author address almost my questions but have the following point if the author can consider it.
1. In the result section what was your criteria to categorize the age of your study participants line number 144(socio-demographic characteristics in the previous question respond rather the author respond about academic position classification of the study participants and he/she can reflect again.

**Answer:** Thank you.

Here in our country, a physician graduated at least at the age of 24( including the intern period), so the lower limit of age taken as 25, and all resident/frontline doctors in government tertiary care hospitals are younger because of a need of in-service training for completion of postgraduation and most of them are usually around 35 years. That is why age was categorized as such. Also, the case records form was structured based on the previous study on burnout among resident doctors(**Reference:** Alhaffar, B.A., Abbas, G. & Alhaffar, A.A. The prevalence of burnout syndrome among resident physicians in Syria. J Occup Med Toxicol 14, 31 (2019). https://doi.org/10.1186/s12995-019-0250-0). And after analysis, the younger age group was found to have a 6.45 times higher chance of burnout than the older age group.

2. In the previous review the authors reflects this Answer: In table -5(High burnout in all three domains in demographic and demographic and Job-related characteristics) - only the variables with p<0.2 in bivariate analysis were included in the multivariable analysis, and in this regard the author is correctly respond and it is acceptable. But still in line number 176 and 177(regression table needs re write correctly such as Factors associated with high burnout in all three Domains… and in this table there are variables such as "Types of employment", <>, that are present in bi-variable analysis but cannot present or cannot appear( eliminated) in multivariable regression model, meaning that have no report of adjusted odds ratio(AOR) either it is significant or insignificant value and when this appear recoding of this variable may be needed, the other point in this table is that writing p- value for COR is not needed( it is better avoid it).

**Answer:**

The Type of employment's P-value was not statistically significant in univariate analysis. That is why multivariate analysis did not calculate the P value for those non-significant variables. However, I remodeled the table according to your suggestions. Please check.

7. PLOS authors have the option to publish the peer review history of their article ([what does this mean?](https://journals.plos.org/plosone/s/editorial-and-peer-review-process#loc-peer-review-history)). If published, this will include your full peer review and any attached files.


If you choose "no", your identity will remain anonymous but your review may still be made public.


**Do you want your identity to be public for this peer review?** For information about this choice, including consent withdrawal, please see our [Privacy Policy](https://www.plos.org/privacy-policy).

Reviewer #3: No

Reviewer #4: No
